# Circulating tumor HPV DNA, antibodies to HPV16 early proteins, and oral HPV16 DNA as biomarkers for HPV-related oropharyngeal cancer screening

**DOI:** 10.1177/18758592241313323

**Published:** 2025-03-21

**Authors:** Kristina R Dahlstrom, Andrew T Day, Victor M Alvarez, Samantha R Chirinos, Giselle Santillana, Ming Guo, Karen S Anderson, Erich M Sturgis

**Affiliations:** 1 Section of Epidemiology & Population Sciences – Department of Medicine, Baylor College of Medicine, Houston, Texas, USA; 2 Department of Otolaryngology-Head and Neck Surgery, The University of Texas Southwestern Medical Center, Dallas, Texas, USA; 3 Department of Otolaryngology-Head and Neck Surgery, Baylor College of Medicine, Houston, Texas, USA; 4 Department of Pathology, The University of Texas MD Anderson Cancer Center, Houston, Texas, USA; 5 Biodesign Institute, Arizona State University, Tempe, Arizona, USA

**Keywords:** Cancer screening, biomarker, human papillomavirus, oropharyngeal cancer, head and neck neoplasms, circulating tumor tissue modified HPV DNA, HPV16 antibodies

## Abstract

**Background:**

Oropharyngeal cancer rates continue to rise with no effective screening method. Persistent oral oncogenic human papillomavirus (HPV), antibodies to HPV16 early (E) oncoproteins, and circulating tumor HPV DNA (ctHPVDNA) are biomarkers that show promise for use in HPV-related cancer screening.

**Objective:**

To assess the prevalence of biomarkers for HPV-related cancer and their agreement in middle-aged men.

**Methods:**

Men aged 50–64 years from the general population provided oral rinse and blood samples as well as information about demographics, tobacco/alcohol exposure, sexual behavior, and HPV-related disease history. Oral rinse was tested for HPV16 DNA and plasma was tested for HPV16 E antibodies and ctHPVDNA using a droplet digital PCR (ddPCR)-based assay that measures circulating tumor tissue modified viral (TTMV)-HPV DNA (NavDx, Naveris, Inc.). We calculated frequency distributions of variables of interest and agreement between the biomarkers.

**Results:**

We enrolled 1045 subjects between April 2017 and April 2024. The 954 subjects with results for all three biomarkers were included in the analysis. The prevalence was 4.9% for oral HPV16 DNA, 0.7% for HPV16 E antibodies, and 0.5% for TTMV-HPV DNA.

**Conclusions:**

The low prevalence of all three biomarkers shows their potential to identify high-risk individuals eligible for further clinical HPV-related cancer screening.

## Introduction

A continued rise in the incidence of human papillomavirus (HPV)-related oropharyngeal cancer (OPC) is expected for the foreseeable future.^1,2^ It is currently not possible to detect precancerous lesions and, due to the nature of the disease, most patients are diagnosed with regionally metastatic disease that requires more aggressive treatment than if diagnosed at earlier stages. If early-stage disease could be identified, minimally invasive surgical techniques could be employed for localized treatment, reducing cancer morbidity and mortality.

Despite the increase in the number of cases, especially in higher-income countries, incidence is still low overall in the general population, although some populations, notably white and middle-aged and older males, and those with high numbers of lifetime sex partners, are at increased risk.^2–4^ Because it is not feasible to screen the entire population for a low-incidence disease, a multi-step approach that consists of identification of a subgroup of high-risk individuals who would benefit from more intensive periodic screening is necessary. Furthermore, confirming high-risk groups for HPV-related cancers could lead to exploration of preventive strategies such as immune modulation and therapeutic vaccines. Detecting premalignant lesions could facilitate testing of therapeutic vaccine approaches.

Serum antibodies to HPV16 early (E) oncoproteins and circulating tumor HPV DNA (ctHPVDNA) are blood-based biomarkers that have been demonstrated to predict risk of HPV-related OPC and recurrence with high accuracy.^5–8^ Another potential marker, persistent oral oncogenic HPV infection, is well-established for cervical and anal cancer screening, but less is known about its potential usefulness for OPC screening.^9^

The integration of these biomarkers into OPC screening protocols holds promise for improving early detection and patient outcomes. Additionally, offering a convenient and non-invasive screening option through the development of home-based testing would be beneficial to individuals that need longer-term monitoring or those living in remote areas. However, further research is needed to evaluate the feasibility of self-sampling and biomarker stability in mailed samples before launching large-scale screening trials. Therefore, the goal of this study was to estimate the prevalence of the three candidate biomarkers and their concordance in a target screening population of middle-aged men taking part in the HOUSTON and TRINITY screening trials for HPV-related cancers.

## Materials and methods

### Experimental subjects

The study population included subjects from the HOUSTON and TRINITY screening trials (ClinicalTrials.gov ID NCT02897427), two population-based studies designed to develop a multi-stage approach to screening for HPV-related cancers through low-cost biomarker evaluation followed by in-person exams for biomarker positive individuals determined to be at increased risk. The HOUSTON study design has been published previously.^10^ Study subjects were males aged 50–64 years without a history of an HPV-related cancer (squamous cell carcinoma of the oropharynx, anus, or penis). Exclusion criteria included receipt of radiation therapy to the head and neck, receipt of active cancer therapy or blood transfusion within the past 6 months, and history of stem cell, bone marrow, or solid organ transplant. For the baseline visit, subjects completed a self-administered questionnaire on demographic characteristics, history of alcohol and tobacco exposure, sexual behaviors, and medical history pertaining to HPV-related diseases. Subjects also provided an oral rinse specimen for oral HPV detection and a blood sample for HPV16 E antibodies and circulating tumor tissue modified viral (TTMV)-HPV DNA testing. The study was approved by the Baylor College of Medicine Institutional Review Board and conforms with The Code of Ethics of the World Medical Association (Declaration of Helsinki). All participants provided written informed consent.

### Laboratory assays

#### Oral HPV16 DNA detection

Ten milliliters of mouthwash (Scope Classic, Procter & Gamble, Cincinnati, OH) was swished in the oral cavity and oropharynx for 15 s, gargled for 15 s, then expectorated into a specimen cup. The sample was centrifuged, washed with PBS, and resuspended in 1 ml PBS. Samples were stored at −80 °C until HPV testing. Prevalent oral oncogenic HPV infection was determined using a commercially available assay (cobas HPV Test, Roche Diagnostics, Indianapolis, IN) that provides HPV type-specific results for types 16 and 18 and a pooled result for 12 other oncogenic types (31, 33, 35, 39, 45, 51, 52, 56, 58, 59, 66, and 68).

#### Plasma HPV16 E antibody detection

Approximately 8 ml of whole blood was collected from each patient in a 10 ml lavender top (EDTA) blood collection tube and processed within 5 h of collection. The sample was centrifuged, and the plasma was stored at −80 °C until testing. For HPV16 E antibody testing, a novel RAPID ELISA was used, the method of which has been published.^11,12^ Briefly, anti-GST antibody was coated onto 96-well plates and blocked using 5% Milk in PBST. GST-tagged proteins were expressed and transferred to the plate, bound, and washed. Human serum or plasma samples were tested at 100-fold dilution.

#### Plasma ctHPVDNA detection

ctHPVDNA was detected using NavDx (Naveris, Inc., Waltham, MA), a clinically validated test that assays ctDNA fragmentation patterns obtained using droplet digital polymerase chain reaction (ddPCR). This assay specifically detects and quantifies circulating TTMV-HPV DNA, and results are reported as the TTMV-HPV DNA Score. This Score, which is adjusted to account for plasma volume, indicates the likelihood a patient has an HPV-associated cancer. Cell-free DNA was isolated from plasma followed by ddPCR to detect and profile TTMV-HPV DNA of oncogenic HPV types 18, 31, 33, and 35.^13^ Positivity was determined using pre-specified thresholds and all runs contained relevant positive and negative controls. Results falling outside the parameters for a positive or negative result were considered indeterminate.

### Statistical analysis

Subjects with results available for all three biomarkers at baseline were included in the analysis. Frequency distributions were tabulated for variables of interest including each biomarker and combination of biomarkers. Samples with an indeterminate TTMV-HPV DNA result were considered positive in binary analysis. Cohen's kappa was used to gauge the level of agreement. P < 0.05 was considered statistically significant, and all tests were two-sided. Stata 16.0 software (StataCorp., College Station, TX) was used for all analyses.

## Results

### Demographic characteristics of the study population

A total of 1045 subjects were enrolled for both studies combined: 553 on the HOUSTON study between April 2017 and December 2019 and 492 on the TRINITY study between December 2021 and April 2024. The 954 subjects with results available for all three biomarkers at baseline were included in the analysis. The demographic characteristics of the cohort are shown in Table 1. The median age for the entire cohort was 57 years (IQR, 54–61) and did not vary by cohort. We observed differences with respect to race, smoking status, annual household income, and marital status (Table 1). The majority of men in both studies were non-Hispanic white (77% overall), but there was a greater proportion of Hispanic participants in the TRINITY study (TRINITY, 27% vs. HOUSTON, 7%; p < 0.001). HOUSTON study participants were more likely to be never smokers, have higher income, and be currently married than TRINITY study participants (p < 0.001 for all), but there was no difference with respect to educational attainment (p = 0.76).

**Table 1.** Demographic characteristics of 954 men aged 50–64 years taking part in the HOUSTON and TRINITY screening trials, overall and by study.

	Total	HOUSTON	TRINITY	
	N = 954	N = 546	N = 408	p-value
Age Group				0.25
50–54	270 (28.3)	150 (27.5)	120 (29.4)	
55–59	370 (38.8)	224 (41.0)	146 (35.8)	
60–64	314 (32.9)	172 (31.5)	142 (34.8)	
Race				<0.001
Non-Hispanic White	726 (76.7)	476 (87.3)	250 (62.3)	
Non-Hispanic Black or African American	38 (4.0)	13 (2.4)	25 (6.2)	
Asian/Pacific Islander	36 (3.8)	18 (3.3)	18 (4.5)	
American Indian/Alaska Native	1 (0.1)	1 (0.2)	0 (0.0)	
Hispanic/Latino	145 (15.3)	37 (6.8)	108 (26.9)	
Smoking status				<0.001
Never	593 (62.8)	372 (68.6)	221 (54.8)	
Former	277 (29.3)	135 (24.9)	142 (35.2)	
Current	75 (7.9)	35 (6.5)	40 (9.9)	
Education				0.76
High school or less	65 (6.9)	35 (6.4)	30 (7.5)	
Some college/College graduate/Technical school	548 (57.9)	320 (58.7)	228 (56.9)	
Advanced degree	333 (35.2)	190 (34.9)	143 (35.7)	
Annual household income				<0.001
Less than $50,000	105 (12.0)	38 (7.4)	67 (18.6)	
$50,000- $99,999	183 (21.0)	101 (19.8)	82 (22.7)	
$100 K or greater	584 (67.0)	372 (72.8)	212 (58.7)	
Marital status				<0.001
Married/Common-law	696 (73.7)	441 (80.8)	255 (64.1)	
Never married	79 (8.4)	33 (6.0)	46 (11.6)	
Separated/Divorced/Widowed	169 (17.9)	72 (13.2)	97 (24.4)	

#### Biomarker test results

The prevalence of all three biomarkers was low (Table 2). Approximately 5% of the full cohort was positive for oral HPV16 DNA at the baseline visit, although it was significantly higher in HOUSTON than TRINITY (7.3% vs. 1.7%, respectively; p < 0.001). The prevalence of HPV16 E antibodies was 0.7% overall but was higher in the HOUSTON cohort compared to the TRINITY cohort (1.1% vs. 0.2%); however, this difference was not statistically significant (p = 0.25). Five participants were positive for TTMV-HPV DNA: two in the HOUSTON study and three in the TRINITY; additionally, five had indeterminate results (p = 0.88).

**Table 2.** Biomarker prevalence among 954 men aged 50–64 years taking part in the HOUSTON and TRINITY screening trials, overall and by study.

	Total	HOUSTON	TRINITY	
Biomarker status	N = 954	N = 546	N = 408	p-value
Oral HPV16 DNA				<0.001
Negative	907 (95.1)	506 (92.7)	401 (98.3)	
Positive	47 (4.9)	40 (7.3)	7 (1.7)	
HPV16 E antibodies				0.25
Negative	947 (99.3)	540 (98.9)	407 (99.8)	
Positive	7 (0.7)	6 (1.1)	1 (0.2)	
TTMV-HPV DNA				0.88
Negative	944 (99.0)	541 (99.1)	403 (98.8)	
Positive	5 (0.5)	2 (0.4)	3 (0.7)	
Indeterminate	5 (0.5)	3 (0.5)	2 (0.5)	

Abbreviations: E: early; HPV: human papillomavirus; TTMV: tumor tissue modified viral

#### Agreement between oncogenic HPV biomarkers

**Figure 1.**
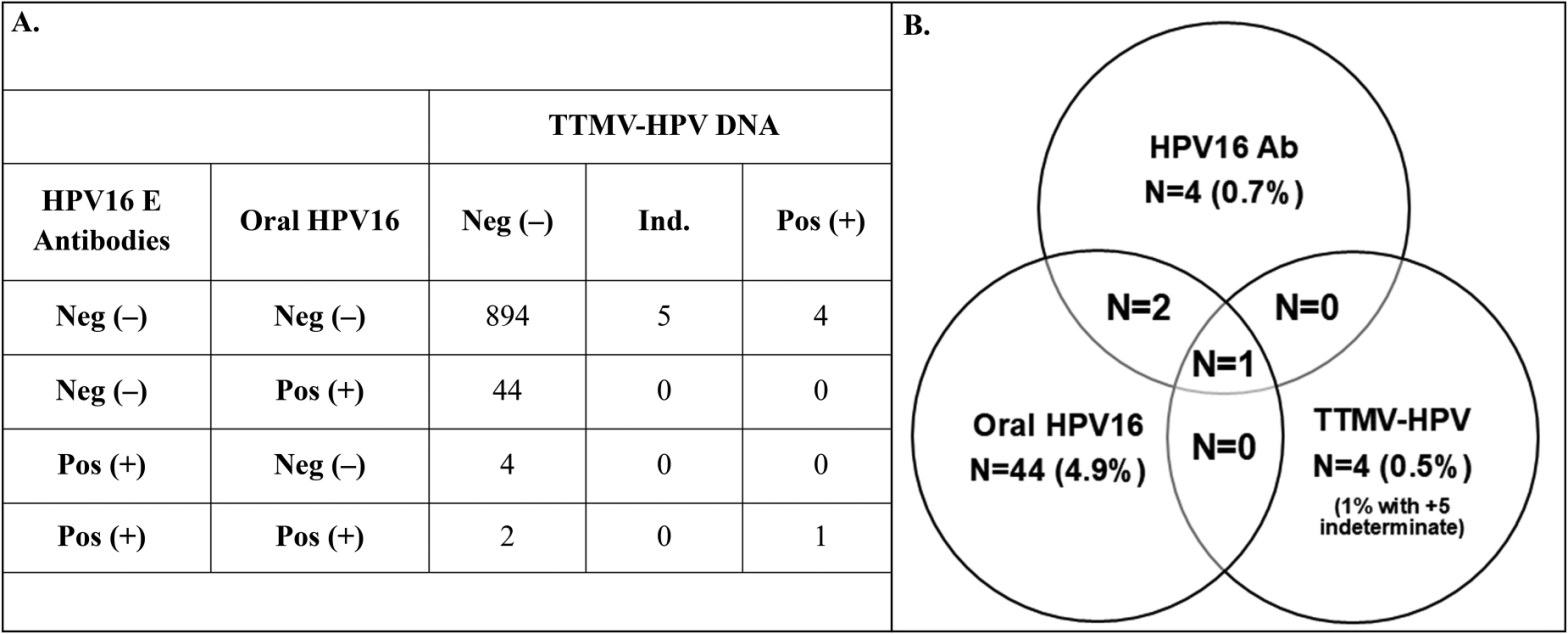
A) Contingency table and B) Venn diagram showing the concordance of oral HPV16 DNA, HPV16 E antibodies, and TTMV-HPV DNA among 954 men aged 50–64 years taking part in the HOUSTON and TRINITY screening trials. Abbreviations: Ab: antibodies; E: early; HPV: human papillomavirus; Ind: indeterminate; TTMV: tumor tissue modified viral.

A contingency table and Venn diagram showing concordance between oral HPV16 DNA, HPV16 E antibodies, and TTMV-HPV DNA is shown in Figure 1. When the five TTMV-HPV DNA indeterminate results were grouped with the positives, concordance between all three markers was high, influenced by the high number of negatives. Sixty men were positive for at least one biomarker (6.3%) with most positive for only one (N = 57; 6.0%), two (0.2%) positive for two, and one (0.1%) positive for all three (Table 3). The agreement between each pair of biomarkers was >94% and Cohen's kappa was statistically significant for the combination of HPV16 E antibodies and oral HPV16 DNA (p < 0.001), TTMV-HPV DNA and HPV16 E antibodies (p < 0.001), and for all three combined (p = 0.001), but was not statistically significant for TTMV-HPV DNA and oral HPV16 DNA (p = 0.228; Table 3).

**Table 3.** Biomarker positivity and agreement for oral HPV16 DNA, HPV16 E antibodies, and TTMV-HPV DNA among 954 men aged 50–64 years taking part in the HOUSTON and TRINITY screening trials.

Number of positive biomarkers by individual	N (%)		
0	894 (93.7)		
1	57 (6.0)		
2	2 (0.2)		
3	1 (0.1)		

Biomarker combination	Percent agreement	κ	p
HPV16 E antibodies-oral HPV16 DNA	95.0%	0.100	<0.001
TTMV-HPV DNA-oral HPV16 DNA	94.2%	0.018	0.228
TTMV-HPV DNA-HPV16 E antibodies	98.4%	0.110	<0.001
Combined κ for All Three Markers		0.057	0.001

Abbreviations: E: early; HPV: human papillomavirus; TTMV: tumor tissue modified viral.

## Discussion

We evaluated the prevalence and concordance of three promising biomarkers for HPV-related OPC screening in the HOUSTON and TRINITY screening cohorts, a target screening population of middle-aged men. As previously reported for the HOUSTON study,^10^ the prevalence of oral HPV16 DNA and HPV16 E antibodies was low in this expanded cohort. Since our last report, we also added TTMV-HPV DNA to our biomarker panel and found a similarly low prevalence. Additionally, among those with a positive result, most were positive for only one marker.

In our study population, the prevalence of oral HPV16 DNA, the primary type detected in OPC tumors, was 5%. Detection of persistent oncogenic HPV DNA in the cervix is a well-established precursor to development of precancer and cancer, and is an essential tool used for cervical cancer screening.^9^ While oral oncogenic HPV is predictive of future HPV-related OPC, current estimates suggest a 40–60-fold increased risk of OPC,^14^ it is unlikely to be useful as a stand-alone screening biomarker as population estimates suggests the prevalence is too high (7% for men and 1.6% for women).^15^ As shown in the HPV in Men study, most infections clear within one year with median time to clearance of 7 months with longer time to clearance for prevalent compared to incident infections.^16,17^ Likewise, in a study of 1833 individuals at-risk for or living with HIV with 10 years of follow-up, about 14% of individuals had a prevalent oral oncogenic HPV infection, almost half of which persisted at least five years with a median of 2.38 years.^18^ Furthermore, of those with a persistent HPV16 infection, two developed an incident OPC during the follow-up period (incidence rate, 1.62 per 100 person-years; 95% confidence interval, 0.41–6.47).^18^ These findings show that repeated measurements to identify long-term persistence would likely be needed to identify a high-risk screening population.

Antibodies to HPV16 E proteins are capable of accurately predicting future development of HPV-related OPC with high sensitivity and specificity as shown by our group and others in case-control studies and the clinical setting.^10,19–22^ In our study population, the prevalence of HPV16 E antibodies was less than 1%, which is consistent with other studies.^21,22^ In an earlier case-control study, we showed a sensitivity of 83% and specificity of 99% for the detection of HPV-positive OPC using a pre-defined algorithm for classification of HPV status.^23^ The European Prospective Investigation Into Cancer and Nutrition cohort and the Prostate, Lung, Colorectal and Ovarian Cancer Screening Trial (PLCO), two large prospective population-based trials, have shown a strong association, with odds ratios > 450, between HPV16 E antibody status and development of OPC several years after a seropositive result.^21,22^ In a meta-analysis, HPV16 E6 seropositivity has a sensitivity and specificity was 83% and 95%, respectively.^24^ Recently, the HPV Cancer Cohort Consortium estimated the risk of developing OPC among those with positive HPV16 E6 serology. The absolute 10-year risks for developing an HPV-related OPC following a positive serologic test at age 50 years and 60 years were 17% and 27% for men, and 4% and 6% for women, respectively.^19^ Furthermore, an E6 seropositive result at age 50 years was predicted to be associated with a lifetime-risk of about 50% for men and 13% for women.

HPV is typically not found in blood during the natural course of an infection and, therefore, detection of ctHPVDNA in plasma suggests an abnormal pathology. Chera et al. has shown that ctHPVDNA can be used as a marker for both disease and risk of recurrence in HPV-related OPC.^25,26^ In a study that included 103 patients with OPC and 55 healthy volunteers, 84 (82%) of the patients had detectable ctHPVDNA prior to treatment while fifty-two (95%) of the controls had no detectable ctHPVDNA, ultimately resulting in a sensitivity and specificity of 89% and 97%, respectively.^25^ Additionally, among the patients, clearance of ctHPVDNA during or after treatment was predictive of improved survival outcomes.^26^ ctHPVDNA is already used in the clinic to monitor patients for recurrence after treatment,^6–8^ but is also a promising option for blood-based screening. This was recently shown by Rettig et al., in a study of archived plasma samples from a hospital-based research biobank.^27^ In samples collected at least 6 months prior to diagnosis, ctHPVDNA was detectable in plasma in 3 of 7 patients who later developed HPV-related OPC, but ctHPVDNA was not detected in the five patients with HPV-negative head and neck tumors or the 100 cancer-free controls.^27^ In a retrospective analysis of hospital biobank samples, a novel HPV whole genome sequencing assay to detect ctHPVDNA had 100% sensitivity and specificity for HPV-related OPC detection up to 4 years before clinical diagnosis with a maximum lead time of 7.8 years.^28^ Maximum lead time was extended to 10.3 years using a Naïve Bayes machine learning model. Comparing ctHPVDNA with oncogenic HPV serology, sensitivity of ctHPVDNA was greater than serology within four years of diagnosis (100% vs. 86%).^28^

Although seropositivity to HPV16 E antibodies is strongly associated with OPC risk, a limitation for its use as a screening biomarker is that detection can precede diagnosis by over a decade. Despite the potential for early intervention, the length of follow-up required may limit compliance over time. Therefore, a biomarker with a shorter median time to diagnosis, such as ctHPVDNA, could serve as an effective addition to a screening algorithm. We found that most biomarker-positive individuals were positive for one marker only. Notably, as we previously reported, the one man who was positive for all three was diagnosed with a HPV16-positive (Epstein-Barr virus-negative) nasopharyngeal cancer shortly after withdrawing from the study (he withdrew before undergoing a clinical exam as part of the study).^10^ The MOUTH study, a population-based study of individuals considered at risk for HPV-related cancer, evaluated the prevalence of oral oncogenic HPV DNA and HPV E6/E7 antibodies and found that 22.4% were positive for at least one oncogenic HPV marker, with 3.7% of individuals positive for an HPV16-specific marker (HPV16 E6 antibodies and oral HPV16 DNA).^20^ This is slightly lower than our finding of 5.4% positivity overall for HPV16 antibodies and oral HPV16 DNA, due to differences in the prevalence of oral HPV16 (4.9% in HOUSTON/TRINITY vs. 1.8% in MOUTH). In the MOUTH study, positivity to HPV16 E6 antibodies was 2.2%, but when other E antibodies were included, as in our binary classifier, prevalence was decreased to 0.2%.^20^ These differences could be explained by differences between the study populations as we only include men and exclude those with a history of HPV-related cancer, the MOUTH cohort includes both men and women with a history of anogenital dysplasia or cancer, or a partner with an HPV-related cancer as well as men with ≥ 2 lifetime oral sex partners.

Our study has some limitations. First, the study population is not representative of men in the general population as most participants were white and of high socioeconomic status. In a new study, we plan to include a more representative population by focusing recruitment on areas outside the immediate Texas Medical Center area where our institution is located. Second, the results presented include the cross-sectional biomarker status at the baseline visit only; therefore, we are unable to draw any conclusions about the association between biomarker status and development of OPC. We plan to present a more detailed clinical picture of the participants we followed over time in a separate publication although our study is not statistically powered to compare incidence of OPC between biomarker-positive and -negative individuals as we expect to detect few HPV-related lesions. The strength of our study is the use of a novel panel of biomarkers with low population prevalence and high sensitivity and specificity for HPV-related OPC as well as a prospective study design with blinded clinical evaluations of biomarker-positive men along with the inclusion of biomarker-negative controls.

## Conclusions

This study found the prevalence of a biomarker panel intended for HPV-related cancer screening was low in a target screening population. Our results confirm earlier findings of low prevalence of these markers in the general population. Used as a first step in screening, a biomarker panel such as this could significantly reduce the number of individuals requiring in-person clinical examinations to detect one case, increasing the cost-effectiveness while limiting the harms associated with screening. Taking advantage of the individual strengths of each of these biomarkers to advance development of a screening paradigm, reducing the burden of HPV-related cancers is possible.
